# Clinical Utility of Skin Biopsy in Differentiating between Parkinson's Disease and Multiple System Atrophy

**DOI:** 10.1155/2015/167038

**Published:** 2015-04-06

**Authors:** Rie Haga, Kazuhiro Sugimoto, Haruo Nishijima, Yasuo Miki, Chieko Suzuki, Koichi Wakabayashi, Masayuki Baba, Soroku Yagihashi, Masahiko Tomiyama

**Affiliations:** ^1^Department of Neurology, Aomori Prefectural Central Hospital, 2-1-1 Higashi-Tsukurimichi, Aomori 030-8553, Japan; ^2^Department of Pathology and Molecular Medicine, Hirosaki University Graduate School of Medicine, 5 Zaifu-cho, Hirosaki 306-8562, Japan; ^3^Diabetes Center, Ohta Nishinouchi Hospital, 2-5-20 Nishinouchi, Koriyama 963-8558, Japan; ^4^Department of Neurophysiology, Institute of Brain Science, Hirosaki University Graduate School of Medicine, 5 Zaifu-cho, Hirosaki 306-8562, Japan; ^5^Department of Neuropathology, Institute of Brain Science, Hirosaki University Graduate School of Medicine, 5 Zaifu-cho, Hirosaki 306-8562, Japan

## Abstract

*Background*. It is often difficult to differentiate Parkinson's disease (PD) from multiple system atrophy (MSA), especially in their early stages. *Objectives*. To examine the clinical utility of histopathological analysis of biopsied skin from the chest wall and/or leg in differentiating between the two diseases. *Methods*. Skin biopsies from the lower leg and/or anterior chest wall were obtained from 38 patients with idiopathic PD (26 treated with levodopa and 12 levodopa-naïve) and 13 age-matched patients with MSA. We sought aggregates of phosphorylated *α*-synuclein on cutaneous nerve fibers using double fluorescence immunohistochemistry and confocal microscopy and measured intraepidermal nerve fiber density (IENFD). *Results*. Phosphorylated *α*-synuclein aggregates were identified on cutaneous nerves in two patients with PD (5.3%) but in none of the patients with MSA, and IENFD was significantly lower in patients with PD when compared to those with MSA. There was no difference in IENFD between levodopa-treated and levodopa-naïve patients with PD. *Conclusions*. Our findings suggest that an assessment of IENFD in biopsied skin could be a useful means of differentiating between PD and MSA but that detection of *α*-synuclein aggregates on cutaneous nerves in the distal sites of the body is insufficiently sensitive.

## 1. Introduction

Parkinson's disease (PD) and multiple system atrophy (MSA) can be differentiated on the basis of neurological symptoms, clinical course, responsiveness to levodopa therapy [1], and imaging findings such as magnetic resonance imaging (MRI) [2] and metaiodobenzylguanidine (MIBG) scintigraphy [3–5]. Nevertheless, in the early stages of both diseases, the symptoms and signs may be similar. Furthermore, although most patients with MSA have little response to levodopa, it has clinical benefits in a small minority [1]. Consequently, more reliable methods are needed to differentiate PD from MSA.

Skin biopsies allow deposits of *α*-synuclein [6–8] and the dermal innervation of patients with PD to be visualized [8, 9]. A postmortem study of patients diagnosed with PD reported that the diagnostic sensitivity and specificity of Lewy pathology in the skin was 70% and 100%, respectively [7]. We previously hypothesized that routine bright-field immunohistochemical identification of *α*-synuclein in biopsied cutaneous tissue could be a useful tool in the premortem diagnosis of PD; however, we could only find Lewy neurites in 10% of skin biopsies taken from patients with a clinical diagnosis of PD [6]. The extent to which Lewy pathology can be detected in the cutaneous nerves of patients with MSA is not known, even though the disease is also caused by abnormal accumulation of *α*-synuclein in the central nervous system [10].

The innervation of the skin is reportedly reduced in patients with PD. A semiquantitative study found reduced dermal sympathetic nerve density in PD compared with controls, suggesting the presence of peripheral autonomic neuropathy in PD [11]. A small study examining dermal sheet preparations found that the presence of small nerve fibers in the dermis is significantly reduced in patients with PD compared with those diagnosed with MSA [12]. Thus, evaluation of nerve density in biopsied skin could be a useful means of differentiating between the two diseases, but the method of preparing dermal sheets has not been standardized. Recently, a reduction in intraepidermal nerve fiber density (IENFD) has been demonstrated in biopsied skin from patients with PD [8, 9]. The quantitative examination of IENFD is well established and standardized [13].

In this study, we examined whether histopathological examination of biopsied skin from patients with PD and MSA could assist in differentiating between the two diseases. We sought evidence of phosphorylated *α*-synuclein on nerve fibers in skin biopsies from patients with PD and MSA, using double fluorescence immunohistochemistry and confocal microscopy to increase sensitivity, and measured IENFD.

## 2. Materials and Methods

### 2.1. Patients

The study was conducted in accordance with the Declaration of Helsinki and was approved by the ethics committee of Aomori Prefectural Central Hospital, Japan. The diagnoses of PD and MSA were based upon the United Kingdom Brain Bank criteria [14] and the Second Consensus Statement [15], respectively. Thirty-eight Japanese patients with idiopathic PD (23 women and 15 men; mean age 64.7 years; mean disease duration 5.8 years) and 13 Japanese patients with probable MSA (six women and seven men; mean age 62.0 years; mean disease duration 3.5 years) were included in the study. They were recruited at the Department of Neurology, Aomori Prefectural Central Hospital, from April 1, 2009, to March 31, 2011. Written informed consent was obtained from each patient after detailed explanation of the procedures and significance of the study. The patients who could not give written informed consent were not included in this study. Of the 38 patients with PD, 26 had previously been treated with levodopa, but 12 were levodopa-naïve. Of the 13 patients with MSA, four had taken levodopa.

Motor impairment was assessed in patients with PD using the Hoehn and Yahr stage and the Unified Parkinson's Disease Rating Scale (UPDRS) Part III 1-2 hours after taking levodopa. We defined the daily levodopa dose as the mean daily dose taken in the last 6 months. Brain MRI or computed tomography had been undertaken in all patients to exclude other diseases presenting with Parkinsonism. Patients older than 75 years and those with a family history of PD were not included in this study. Patients in whom glycated hemoglobin exceeded 6.1% or patients who were deficient in folate (serum concentration < 3.6 ng/mL), vitamin B1 (serum concentration < 20 ng/mL), or vitamin B12 (serum concentration < 233 pg/mL) or who were with abnormal motor and sensory nerve conduction in the tibial and sural nerves [16] were excluded. We also excluded patients with abnormal responses to examination with a pin in the distal extremities or abnormal vibration sensation (<10 s) at the medial malleolus. The clinical and demographic characteristics of the participants are summarized (Table 1).

### 2.2. Skin Biopsy

Under local anesthesia with 1% xylocaine, 6 mm punch biopsies, including the dermis and subcutaneous fat, were obtained from the chest wall (central precordium) and the lower limb (10 cm above the lateral malleolus). Twelve patients (10 with PD and two with MSA) declined to undergo chest wall biopsy but consented for skin to be taken from the lower limb. Specimens from the chest wall and leg were used for immunohistochemical examination; tissue from the leg was used for evaluation of nerve fiber density. Specimens were prepared for double immunofluorescence immunohistochemistry to detect *α*-synuclein aggregates and measure IENFD.

### 2.3. Double Immunofluorescence Immunohistochemistry

Biopsied skin was fixed in Zamboni solution at 4°C overnight, stored in cryoprotected phosphate buffer with 20% sucrose overnight, and then frozen for later cryosectioning.

Skin samples were cut perpendicularly into 60 *μ*m thick sections and immunostained as previously described [17]. The presence of *α*-synuclein on nerve fibers was identified by double staining with mouse monoclonal primary antibodies against protein gene product- (PGP-) 9.5 (a pan-neuronal marker) (1 : 4000, Ultraclone, Isle of Wight, UK) and antiphosphorylated *α*-synuclein (1 : 4000, WAKO, Osaka, Japan) reported with DyLight 549- and DyLight 488-labeled secondary antibodies (1 : 400, Jacskson ImmunoResearch, West Grove, PA, USA). Similarly, PGP-9.5 (1 : 1000), a rabbit polyclonal primary antibody against mouse type IV collagen (1 : 1000, Millipore, Bedford, Oregon, USA), and Alexa 546- and Alexa 488-labeled secondary antibodies (Molecular Probes, Eugene, Oregon, USA) were used to determine IENFD. Sections were mounted on coverslips with the Prolong Gold antifade reagent and 4′,6-diamidino-2-phenylindole (DAPI, Molecular Probes) and imaged using a confocal microscope (Zeiss LSM510, Carl Zeiss, Oberkochen, Germany) with a 40× water immersion objective lens and appropriate filters at a resolution of 1024 × 1024 pixels. Sixteen confocal images were captured at 2 *μ*m intervals and analyzed using proprietary image browser software (Carl Zeiss). Four skin sections of subcutaneous tissue from each patient were used for *α*-synuclein detection. To measure the IENFD, eight to 10 fields from at least two different sections from each patient were collected for analysis. The IENFD was defined as previously described [17]. Briefly, nerve fibers penetrating the basement membrane labeled by anti-type IV collagen were counted by an investigator blinded to the patients' diagnoses.

### 2.4. Statistical Analysis

We used Student's* t*-test, Spearman's rank correlation, and the Mann-Whitney test to examine the relationships between IENFD and levodopa dose, diagnosis and UPDRS, and diagnosis and IENFD. Values are expressed as mean ± standard deviation, and statistical significance was set at *P* < 0.05. All analyses were undertaken with the Ekuseru-Toukei 2008 computer software program (Social Survey Research Information Co., Ltd., Japan). One-sided Fisher's exact test was performed to analyze association between *α*-synuclein deposits on skin nerves and the diagnosis of PD.

## 3. Results

### 3.1. Accumulation of Phosphorylated *α*-Synuclein in the Skin

**Figure 1:**
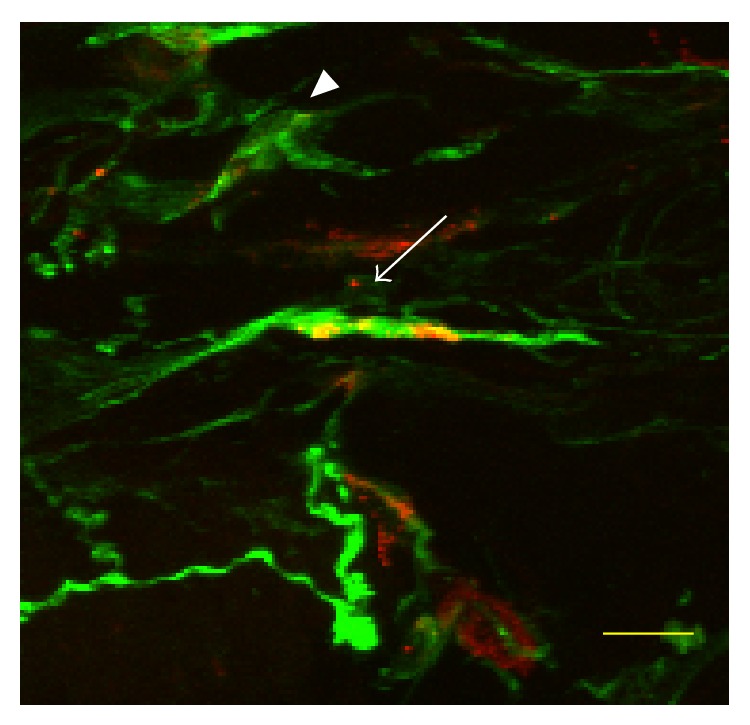
Phosphorylated *α*-synuclein accumulation in the skin nerve fiber. Confocal microscopic colocalization study of phosphorylated *α*-synuclein deposits (red) in skin biopsied from the lower limb of a patient with Parkinson's disease. The pan-axonal marker PGP-9.5 in green reveals nerve fibers (arrow) and fibroblasts (arrowheads). Colocalization (yellow) is observed in a subcutaneous nerve fiber. Bar = 10 *μ*m.

The double immunofluorescence method revealed abnormal *α*-synuclein deposits on the nerve fibers in two of 38 patients with PD (5.3%, Figure 1). One patient was a 71-year-old woman with 4-year disease duration (Hoehn and Yahr stage 3 and UPDRS 26). The staining was identified in the skin biopsy taken from the leg. The other patient was a 70-year-old man with a 1-year history of disease (Hoehn and Yahr stage 2 and UPDRS 24), in whom immunopositive staining was found in the chest wall skin biopsy specimen. The average of disease duration of PD patients without *α*-synuclein deposits on the nerve fibers was 4.8 ± 3.2 years. We could not find significant relationship between detection of *α*-synuclein and the diagnosis of PD (*P* = 0.55, one-sided Fisher's exact test). No *α*-synuclein staining could be found in any of the biopsy specimens from patients with MSA.

### 3.2. Intraepidermal Nerve Fiber Density

**Figure 2:**
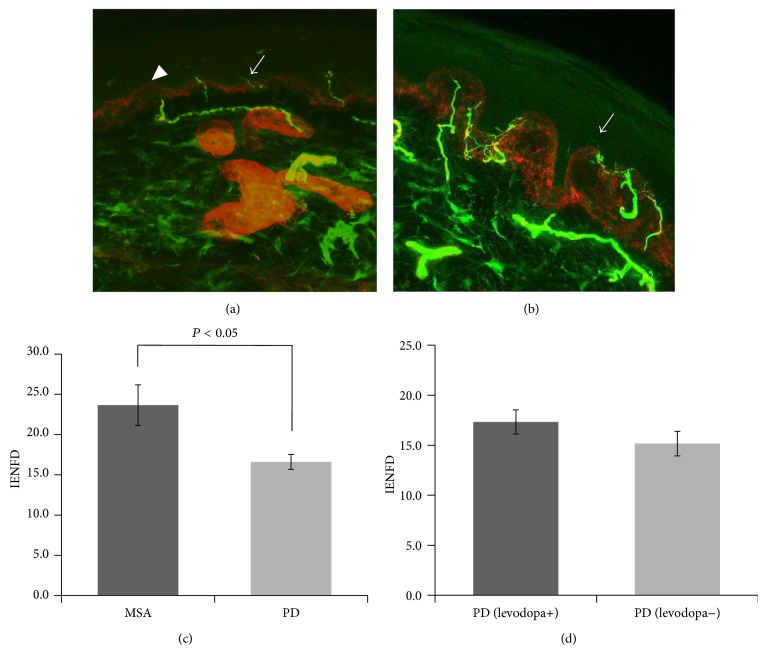
Intraepidermal nerve fiber density in patients with multiple system atrophy (MSA) and patients with Parkinson's disease (PD). ((a) and (b)) Confocal digital images showing a decrease in intraepidermal nerve fiber density in Parkinson's disease (a) compared with multiple system atrophy (b). Nerve fibers and fibroblasts are labeled by PGP-9.5 (in green) and the basement membrane of the skin is labeled by collagen IV (in red; arrowheads). The number of nerve fibers penetrating the basement membrane (arrows) was quantified. (c) Intraepidermal nerve fiber density in patients with multiple system atrophy (MSA) and patients with Parkinson's disease (PD). Fiber density is expressed as the number of counted nerves per linear millimeter at the basement membrane. Fiber density was significantly decreased in patients with PD compared with those with MSA. Error bars show standard error of the mean. (d) Intraepidermal nerve fiber density in patients with PD exposed to levodopa (levodopa+) and those naïve to levodopa (levodopa−). No significant difference was observed between two groups. IENFD: intraepidermal nerve fiber density; MSA: multiple system atrophy; PD: Parkinson's disease.

Confocal microscopy showed that penetration of the basal membrane of the skin by nerve fibers was diminished in patients with PD (16.6 ± 0.9 fibers/mm, mean ± SEM) compared with those with a diagnosis of MSA (23.7 ± 2.5 fibers/mm, Figures 2(a) and 2(b)), reflected by reduced IENFD (Figure 2(c)). There was no significant difference in IENFD between patients with PD previously treated with levodopa (17.3 ± 1.2 fibers/mm) and those who were levodopa-naïve (15.2 ± 1.2 fibers/mm, Figure 2(d)). The IENFD of two PD patients with *α*-synuclein deposits on nerve fiber were 20.5 and 13.0 fibers/mm. On the other hand, the average of PD patients without *α*-synuclein deposits was 16.7 ± 1.0 fibers/mm. No tendency was drawn from the IENFD difference between patients with and without *α*-synuclein deposits.

## 4. Discussion

We examined whether skin biopsy is a clinically useful means of differentiating between PD and MSA. We found that (1) even detailed immunohistochemical examination failed to detect phosphorylated *α*-synuclein deposits in skin specimens taken from most patients with PD; (2) *α*-synuclein deposits were not detected in skin biopsies taken from patients with MSA; (3) sensory IENFD was reduced in patients with PD compared with those with MSA; and (4) there was no difference in the IENFD in patients previously treated with levodopa and those who had not been exposed to the drug. Taken together, immunoreactivity to phosphorylated *α*-synuclein may not be a reliable means of establishing a diagnosis in patients suspected to have either PD or MSA, but measurement of IENFD may have a role to play.

Ikemura et al. used routine immunohistochemistry of autopsy specimens to show that the sensitivity of Lewy pathology in the skin for a diagnosis of PD was 70% [7]. However, in our previous biopsy study we had only found Lewy neurites in two of 20 patients (10%) with clinically diagnosed PD [6]. This discrepancy could be explained by differences in tissue sampling. Lewy neurites in the skin are often found in nerve bundles in subcutaneous tissue [7]. Obtaining skin at autopsy allows deeper subcutaneous structures and larger specimens to be taken compared with skin biopsy.

We used double immunofluorescence histochemistry against phosphorylated *α*-synuclein to try to improve the sensitivity of Lewy pathology detection in the skin of patients with PD. This technique may enable detection of *α*-synuclein accumulation even on small diameter nerve fibers. Despite this approach, only two of 38 patients (5.3%) diagnosed with PD in our series had deposits immunoreactive for the monoclonal antibody. It may be therefore reasonable to conclude that the sensitivity of detecting Lewy neurites in biopsied skin is too low to be a clinically useful biomarker of PD. However, Doppler et al. [18] have recently demonstrated that deposits of phosphorylated *α*-synuclein in biopsied skin were identified in 16/31 PD patients but in 0/35 controls. They concluded that detection of phosphorylated *α*-synuclein in dermal nerve fibers might be a useful diagnostic test for PD. It was of note that they took skin specimens from the back, proximal and distal leg, and finger. Phosphorylated *α*-synuclein deposition was more frequently found at more proximal biopsy sites. We took skin from the distal leg and anterior chest wall, indicating that we analyzed *α*-synuclein deposits in the most distal sites of the peripheral nervous system. The difference in the site of skin biopsy may account for a difference in sensitivity. The site of skin biopsy is another matter to be debated.

Nonetheless, the specificity of positive *α*-synuclein staining in skin nerve fibers for a diagnosis of PD is high [18]. Recently, a double immunofluorescence histochemical study reported that *α*-synuclein deposition is increased in cutaneous sympathetic nerve fibers in skin biopsied from patients with PD compared with controls [8]. The techniques for fixation and staining used by Wang and colleagues are similar to ours, although they used a polyclonal antibody to *α*-synuclein while we used a monoclonal antibody against phosphorylated *α*-synuclein, highly specific for the abnormal *α*-synuclein that accumulates in Lewy neurites. Nonspecific *α*-synuclein antibodies will bind both normal and abnormal *α*-synuclein, and immunopositive staining with the polyclonal antibody can be identified not only on nerves from patients with PD but also on nerves from normal controls [8]. Their results were adjusted by normalizing the amount of deposits to the nerve fiber density by calculating the *α*-synuclein ratio. It is important to note that the binding of *α*-synuclein by the nonspecific polyclonal antibody is increased in autonomic peripheral nerves in PD [8] and the analysis of the *α*-synuclein ratio may be valuable in the diagnosis of PD. However, normal *α*-synuclein may accumulate in axons when axonal flow is disturbed [19]. As axonal flow is thought to be disturbed in PD [20], but also in a variety of other diseases, it is possible that the accumulation of *α*-synuclein recognized by the nonspecific antibody is not necessarily pathognomonic. Although the *α*-synuclein burden appears to be increased in the peripheral nervous system in PD, the clinical significance of Wang and colleagues' findings is still a matter of debate. To date, there have been no reports that *α*-synuclein is deposited on the skin in MSA: none of the patients with MSA were found to have accumulation of abnormal *α*-synuclein in our cohort. This is logical as the peripheral autonomic nervous system is preserved in MSA [21]. In contrast, however, abnormal *α*-synuclein deposits are reportedly found in the skin of patients with pure autonomic failure (PAF): indeed it was a patient with PAF in whom Lewy neurites were first described in biopsied skin [22]. More recently, it has been reported that all patients with PAF have evidence of phosphorylated *α*-synuclein deposition in cutaneous sympathetic nerve fibers [21]. This suggests that seeking neuritic inclusions of phosphorylated *α*-synuclein in the skin could provide a sensitive biomarker for PAF. We found aggregates immunoreactive for phosphorylated *α*-synuclein in the skin of few patients with PD. The density of neuronal axons in the skin might, however, be substantially diminished in PD, in the same way that distal-dominant damage occurs in cardiac sympathetic nerves in the disease [20]. That is, peripheral nerves in the skin of distal sites may have been so severely affected in PD that phosphorylated *α*-synuclein deposits might be found in few PD patients. Furthermore, drug-naïve PD patients had no such deposits on cutaneous nerves, suggesting that peripheral nerves may be damaged even in the early stages of PD as previously described [18].

Detection of epidermal nerve fibers provides standardized objective means of assessing the density of distal nerve endings of small myelinated A-delta and unmyelinated C primary afferent sensory fibers [22]. Small myelinated A-delta afferents lose their myelin sheaths when they penetrate the basal membrane. Thus, all epidermal nerve fibers are unmyelinated. This phenomenon has diagnostic value in patients with small fiber neuropathy, in whom sensory symptoms and pain occur in the distal extremities but standard nerve conduction studies fail to show abnormalities [13]. It has been shown that the IENFD is reduced in skin biopsied from patients with PD [19] and MSA [21] when compared with controls. An abnormal IENFD may be a consequence of the reduction in unmyelinated nerve fibers in the sural nerve in PD and MSA [23]. Here, we showed that IENFD was significantly decreased in PD compared with MSA (Figure 2). This concurs with Novak and colleagues, who reported the same findings in the dermis of skin sheets prepared from patients with PD and MSA [12] but who did not use the standardized IENFD technique [17]. Our result suggests that analysis of IENFD might help to differentiate between PD and MSA.

There is accumulating evidence that levodopa exposure is a risk factor for the development of sensory neuropathy in PD [24–26]. It appears that levodopa reduces serum vitamin B12 concentration and increases serum homocysteine concentration [24, 26] but this issue is still controversial [18]. In our cohort, the duration of treatment and doses of levodopa were both greater in patients with PD than patients with MSA (Table 1). However, IENFD was also similarly reduced in levodopa-naïve patients with PD (Figure 2(d)), supporting the hypothesis that the reduction in IENFD is a consequence of PD rather than levodopa treatment [18]. We excluded a small number of patients with PD and clinical neuropathic signs from our study, so our findings may not reflect the pathophysiology of levodopa-induced neuropathy.

It remains to be determined whether sensory nerve denervation in the skin of PD patients stems from *α*-synuclein deposition. Donadio et al. found that the density of somatic and autonomic skin fibers was reduced in patients with PAF [21] with autonomic fibers staining for phosphorylated *α*-synuclein but not sensory nerve fibers. They concluded that abnormalities in somatic innervation in PD may constitute secondary damage induced by tissue changes caused by dysautonomia [21]. Although phosphorylated *α*-synuclein deposits were not found throughout the dorsal root ganglia of patients with PD [27], there is evidence of positive immunostaining in the lateral portion of the dorsal root, consisting of unmyelinated and thinly myelinated fibers [28]. It is therefore possible that the reduction in IENFD results from direct damage by *α*-synuclein accumulation in sensory nerves.

There is a limitation in the present study because this lacked disease-free controls. Punch biopsy of the skin is to some extent invasive to patients. Then, we needed to minimize subjects' distress and took precedence to accomplish the minimum purpose to examine whether histopathological examination of biopsied skin from patients with PD and MSA could assist in differentiating between the two diseases. We could firstly demonstrate a decrement of IENFD in PD patients compared with MSA patients using standardized technique [17]. The idea that IENFD measurement in biopsied skin is a useful tool for diagnosis of PD would be confirmed when larger studies are conducted.

## 5. Conclusions

The measurement of IENFD in leg skin specimens would be clinically helpful in differentiating between PD and MSA. However, it may not be feasible to differentiate between PD and MSA by detecting *α*-synuclein deposits on nerves in biopsied skin taken from the distal sites in view of the peripheral nervous system. More proximal sites of the body may be appropriate to be biopsied to detect *α*-synuclein deposits in the skin.

## Figures and Tables

**Table 1:** Clinical characteristics of patients with Parkinson's disease and multiple system atrophy.

	MSA	PD		
		Total	Levodopa(+)	Levodopa(−)
	(*n* = 13)	(*n* = 38)	(*n* = 26)	(*n* = 12)
Age (yrs)	62.7 ± 8.4	64.1 ± 5.7	64.5 ± 5.5	63.1 ± 6.2
Duration (yrs)	3.0 ± 1.1	4.8 ± 3.2	5.4 ± 3.4	3.2 ± 1.9^†^
UPDRSIII	—	20.7 ± 11.3	20.4 ± 12.5	21.3 ± 8.2
Hoehn and Yahr	—	2.4 ± 0.7	2.5 ± 0.7	2.3 ± 0.6
Levodopa dose	92 ± 178	222 ± 177^∗^	324 ± 109	0

Data are expressed as the mean ± standard deviation. Levodopa dose was the mean daily dose taken in the last 6 months before skin biopsy.

^∗^PD versus MSA: *P* < 0.01.

^†^PD levodopa(+) versus PD levodopa(−): *P* < 0.01.

MSA: multiple system atrophy; PD: Parkinson's disease; PD levodopa(+): Parkinson's disease with exposure to levodopa; PD levodopa(−): Parkinson's disease naïve to levodopa; UPDRS-III: Unified Parkinson's Disease Rating Scale.
